# Supplementary material for: Adaptive and Resilient Soft Tensegrity Robots
Source: Soft Robot. 2018 Jun 1;5(3):318–29. doi: 10.1089/soro.2017.0066 (PMC6001847; doi:10.1089/soro.2017.0066)
Supplement: Supplemental data [file Supp_Video1.zip › Supp_Video1.pdf]

Supplementary Data

SUPPLEMENTARY VIDEO S1. Presentation of our soft tensegrity robot. The video shows the soft tensegrity robot in action: how it can locomote and how it can learn to compensate when damaged.
